# Thermalization in a Quantum Harmonic Oscillator with Random Disorder

**DOI:** 10.3390/e22080855

**Published:** 2020-07-31

**Authors:** Ya-Wei Hsueh, Che-Hsiu Hsueh, Wen-Chin Wu

**Affiliations:** 1 Department of Physics, National Central University, Jhong-li 32001, Taiwan; yhsueh@phy.ncu.edu.tw; 2 Department of Physics, National Taiwan Normal University, Taipei 11677, Taiwan

**Keywords:** thermalization, quantum harmonic oscillator, random disorder

## Abstract

We propose a possible scheme to study the thermalization in a quantum harmonic oscillator with random disorder. Our numerical simulation shows that through the effect of random disorder, the system can undergo a transition from an initial nonequilibrium state to a equilibrium state. Unlike the classical damped harmonic oscillator where total energy is dissipated, total energy of the disordered quantum harmonic oscillator is conserved. In particular, at equilibrium the initial mechanical energy is transformed to the thermodynamic energy in which kinetic and potential energies are evenly distributed. Shannon entropy in different bases are shown to yield consistent results during the thermalization.

## 1. Introduction

Microscopic description of the thermalization has been a longstanding question in isolated quantum systems. The biggest enigma is due to the discordance between reversible microscopic laws and irreversible macroscopic phenomena. Among many fundamental issues, one common question is whether an isolated quantum system can reach thermal equilibrium, i.e., the state with maximum entropy [1,2,3,4,5,6,7,8]. Fortunately, ultracold quantum gases, which are both pure and controllable, provide an excellent platform to study the nonequilibrium dynamics for isolated quantum systems. In atomic Bose–Einstein condensate (BEC) experiments, Kinoshita et al. [9] showed no evidence of thermalization by pairwise collision, from the Tonks–Girardeau limit to the intermediate coupling regime. However, the dissipative motion of oscillating BEC in a disordered trap, done by Dries et al. [10], did manifest the thermalization [11].

The phenomenon is even more interesting as it relates to Anderson localization (AL)—one of the most important topics in condensed-matter physics [12]. AL has recently been observed in various systems such as discrete-time quantum walks [13], Rydberg electrons [14], photonic lattices [15], monolayer graphene [16], and even quantum chormodynamics [17,18]. Since 2003, AL has been extensively studied and realized in BEC with random disorder both experimentally [19,20] and theoretically [11,19,20,21,22,23,24,25,26,27,28,29,30,31,32,33,34]. These works considered AL in BEC in various regimes and setups. More references can be found in the review article of Sanchez-Palencia et al. [35].

The quantum harmonic oscillator is one of the simplest systems in physics and plays a central role in a wide variety of fields [36]. For example, quantum harmonic oscillator appears everywhere in quantum optics and its properties have been seen regularly in experiments. In ultracold atoms, one can use the Feshbach resonance technique to tune the *s*-wave scattering length to zero [37]. Inspired by the experiment of an oscillating BEC in disordered trap [10], we aim to study the thermalization in a disordered quantum harmonic oscillator. It will be shown in this system that the reversible microscopic quantum mechanics actually conceals the irreversible macroscopic phenomena of thermodynamics.

As mentioned earlier, works from over a decade ago have already discussed the related phenomena in disordered BEC in the mean-field regime [19,20,30,31,32,33,35]. We nevertheless try to present an exact result for a “noninteracting” quantum oscillator in a disorder trap. In principle, the exact noninteracting harmonic oscillator could still be very different from the disordered dipole-oscillating BEC in the mean-field regime, even in the noninteracting limit. Our results show that equilibrium properties are very different between the current disordered noninteracting quantum oscillator and the disordered dipole-oscillating BEC in the mean-field regime [38]. More interestingly, it will be shown in the current quantum system that the equilibrium properties are very similar to those for a classical harmonic oscillator in the microcanonical ensemble. The kind of quantum-to-classical transition indicates strongly the “intrinsic decoherence” of the isolated system [39,40].

A possible scheme to this problem will first be proposed. In the theoretical simulation, we alternatively prepare an initial coherent state with a centroid velocity $v_{0}$, which is an out-of-equilibrium state. Owing to the multiple scattering with the disorder, more and more (initial) mechanical energy will transform to the thermodynamic one. Once the mechanical energy is fully transformed to the thermodynamic one, the system reaches the equilibrium. During the whole process from nonequilibrium to equilibrium, total energy is conserved. Moreover, at equilibrium, total energy is evenly distributed between the kinetic energy and the potential energy associated with harmonic trap.

The paper is organized as follows. In Section 2, we propose the possible scheme to study the thermalization in a quantum harmonic oscillator with random disorder. Theoretical approach for the simulation is also introduced. In Section 3, we show the results of real-space density and momentum distributions when the system reaches the equilibrium. In Section 4, due to the effect of disorder, we show that mechanical energy is transformed to the thermodynamic one. In addition, at equilibrium thermodynamic energy is evenly distributed between kinetic energy and harmonic potential energy. In Section 5, Shannon entropy in different bases are studied for the thermalization process. Section 6 is a conclusion.

## 2. The Approach

To study the thermalization of a quantum harmonic oscillator with random disorder, our approach is to solve the time-evolution wavefunction ψ of the particle for the whole process from nonequilibrium to equilibrium. The corresponding properties can be accessed based on the solved ψ.

**Figure 1:**
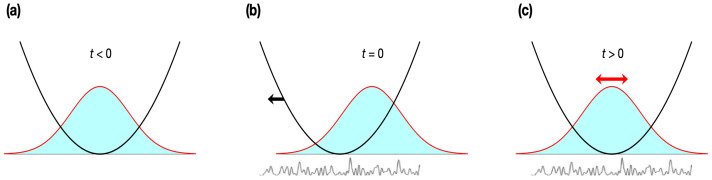
Schematic of the setup for a quantum harmonic oscillator with random disorder. (**a**) At t<0, the disorder is off and the system is in the ground state with a Gaussian wavefunction. (**b**) At t=0, the trap is abruptly displaced to the left, so the system gains an energy to move to the left. Disorder is on at this point. (**c**) At t>0, the system oscillates left and right until it comes to equilibrium due to the disorder effect.

For simplicity, we consider a one-dimensional quantum harmonic oscillator in a disordered trap. The time-dependent Schrödinger equation (TDSE) describing the system is

(1) $$i\hbar \partial_{t}\psi \left(x,t\right)=\left[-\frac{\hbar^{2}\partial_{x}^{2}}{2m}+\frac{1}{2}m\omega^{2}x^{2}+V_{\mathrm{dis}}\left(x\right)\right]\psi \left(x,t\right),$$

where *m* is the particle mass, ω is the trapping frequency, and $V_{\mathrm{dis}}\left(x\right)$ is the Gaussian correlated disorder potential which satisfies the autocorrelation function

(2) $$\int_{-\infty }^{\infty }V_{\mathrm{dis}}\left(x\right)V_{\mathrm{dis}}\left(x+\Delta x\right)dx=V_{D}^{2}\exp\left(-2\Delta x^{2}/\sigma_{D}^{2}\right).$$

where $V_{D}$ and $\sigma_{D}$ correspond to the strength and correlation length of the random disorder, respectively. Figure 1 illustrates the set-up of a possible experiment. Before the disorder potential is turned on, the system is in the ground state with the Gaussian wavefunction. At t=0, the trap is abruptly displaced to the left, so the system gains energy and starts to move to the left. Afterwards, it oscillates. Note that at the same (t=0), the disorder potential is turned on and due to the effect of it, the system will eventually come to equilibrium.

Alternatively, for convenience in simulation, at t=0 an initial coherent state with a velocity $v_{0}$ at the center is prepared for the system,

(3) $$\psi \left(x,0\right)=\psi_{g}\left(x\right)\exp\left(imv_{0}x/\hbar \right),$$

where $\psi_{g}\left(x\right)={\left(m\omega /\pi \hbar \right)}^{1/4}\exp\left(-m\omega x^{2}/2\hbar \right)$ is the Gaussian ground state. After release, owing to the multiple scattering from the disorder, the wave packet starts to redistribute. When evolution time is long enough ($t\gg t_{\mathrm{th}}$, $t_{\mathrm{th}}$ is the thermalization time defined in Section 5), and the system approaches the equilibrium. Together with (2) and (3), we have numerically solved (1) for a long enough time to see the phenomenon of thermalization. In our simulation, $\varepsilon_{0}\equiv \hbar \omega$ and the trapping length $l_{0}\equiv \sqrt{\hbar /m\omega }$ are taken as the units of energy and length.

Here, we detail the simulation method to solve the TDSE (1). We performed the fast Fourier transform (FFT) to calculate the integration and differentiation in space. The calculation domain is fixed at [−64π,64π] with a grid of N=12,288 points. Moreover, the time integration is done by adaptive Runge–Kutta method of orders 4 and 5, built-in in Matlab software. The random Gaussian correlated disorder potential is taken as $V_{\mathrm{dis}}\left(x\right)=V_{D}f\left(x\right)$, where

(4) $$f\left(x\right)=\sum_{i=1}^{N}A_{i}\exp\left[-\frac{4{\left(x-x_{i}\right)}^{2}}{\sigma_{D}^{2}}\right]$$

with $x_{i}$ the grid points. The generated random numbers $A_{i}$ are subject to $\langle A_{i}\rangle =0$ and $\langle A_{i}^{2}\rangle =1$, and the normalization $\int_{-\infty }^{\infty }f{\left(x\right)}^{2}dx=1$ is finally made to satisfy Equation (2). We have been assured that the wave function has not been contaminated by numerical noise (round-off error) even after long-time computation. One can also consider the non-Gaussian correlated disorders such as the sinc(x)=sinx/x correlated function considered in [30,34]. We have also done the calculation based on the sinc correlated disorder function and the results are found to be the same as the presented ones. It is also worth noting that all results obtained are from a single (fixed) random potential. The result does not average over noise at any points.

## 3. Equilibrium Distribution

It is important to note that “equilibrium” has a precise meaning in thermodynamics, namely, the systems is described with a Boltzmann distribution when it is coupled with a reservoir at temperature *T*. There is no “reservoir” in the current isolated system. Here, “equilibrium” means a state of balance or a stable situation in which different parts of energies will no longer exchange internally. As will be seen clearly, disorder plays the role to assist the energy exchange and lead to the “equilibrium” of the system. In this regard, the thermalization discussed in the present paper can be viewed as a kind of “intrinsic thermalization”.

It is also important to note that a system connected to a random disorder cannot in general be regarded as an isolated one. The isolated system under consideration refers to the combination of the harmonic oscillator and the random disorder. The real and time-independent random Gaussian correlated disorder considered does fulfill the time reversibility of Equation (1) and, at the same time, results in the maximization of Shannon entropy (see Section 5).

With the solved dynamic wavefunction ψ(x,t), one is ready to see the real-space density distribution

(5) $$\rho \left(x,t\right)={\left|\psi \left(x,t\right)\right|}^{2}.$$

At t=0 before release, the real-space density distribution is given by

(6) $$\rho \left(x,0\right)={\left|\psi \left(x,0\right)\right|}^{2}={\left(m\omega /\pi \hbar \right)}^{1/2}\exp\left(-m\omega x^{2}/\hbar \right).$$

Once the system starts to oscillate, due to multiple scattering with the random disorder, the system will eventually reach equilibrium from nonequilibrium.

**Figure 2:**
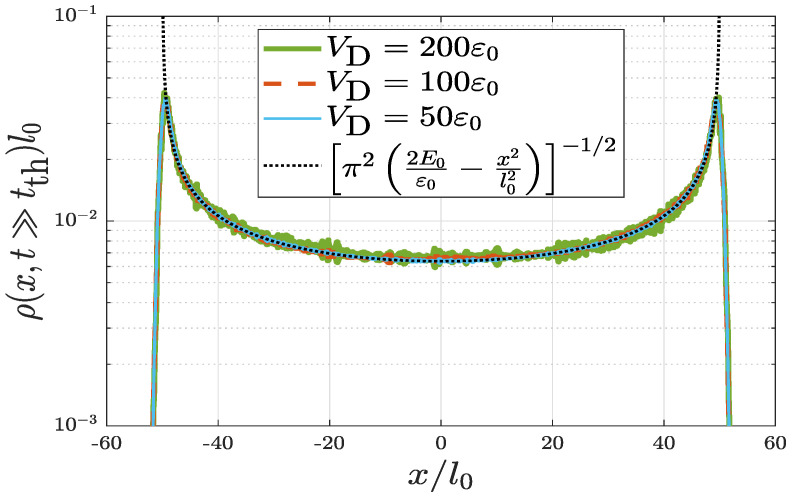
Equilibrium real-space density distributions $\rho (x,t\gg t_{\mathrm{th}})$ for a quantum harmonic oscillator with random disorder. Three cases of $V_{D}=50\varepsilon_{0}$ (solid blue curve), $100\varepsilon_{0}$ (dotted red curve), and $200\varepsilon_{0}$ (dot-dashed green curve) are shown which have almost overlapped with each other. Parameters $\sigma_{D}=0.01l_{0}$ and $v_{0}=50l_{0}\omega$ are fixed in all three cases. For comparison, dashed black curve corresponds to the distribution of a classical harmonic oscillator in a microcanonical ensemble (see also Equation (8)).

Figure 2 shows the equilibrium real-space density distributions at $t\gg t_{\mathrm{th}}$. Three cases of disorder strength $V_{D}=50\varepsilon_{0}$, $100\varepsilon_{0}$, and $200\varepsilon_{0}$ are shown. The disorder correlation length $\sigma_{D}=0.01l_{0}$ and the initial velocity $v_{0}=50l_{0}\omega$ are fixed for all three cases. As shown, the three equilibrium distributions have almost overlapped with each other. It means that different finite disorder potential strengths lead to the same equilibrium distribution for the isolated quantum oscillator. Of equal importance, the distribution is very close to the microcanonical distribution for a classical harmonic oscillator. In a microcanonical ensemble of a classical harmonic oscillator with a given energy $E_{0}$, the probability distribution in phase space is

(7) $$\rho_{c}\left(x,p\right)=\frac{1}{2\pi \hbar }\delta \left(H-E_{0}\right)\;;\;\;\;H=\frac{p^{2}}{2m}+\frac{1}{2}m\omega^{2}x^{2}$$

and thus the corresponding real-space density distribution is

(8) $$\rho_{c}\left(x\right)=\int_{-\infty }^{\infty }dp\;\rho_{c}\left(x,p\right)=\frac{1}{\pi l_{0}\sqrt{\frac{2E_{0}}{\varepsilon_{0}}-\frac{x^{2}}{l_{0}^{2}}}}.$$

The singularities at the classical turning points correspond to $E_{0}=mv_{0}^{2}/2=1250\varepsilon_{0}$. The transition from a quantum distribution to a classical one provides a clear evidence of quantum (intrinsic) decoherence for the current system.

**Figure 3:**
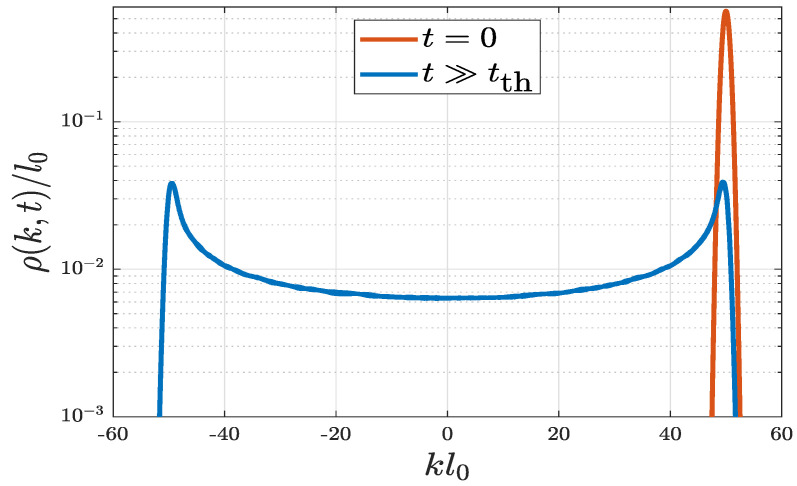
Equilibrium momentum distributions ρ(k,t) for a quantum harmonic oscillator with random disorder. Red and blue curves correspond to the distribution at t=0 (nonequilibrium) and $t\gg t_{\mathrm{th}}$ (equilibrium), respectively. Parameters used are $V_{D}=50\varepsilon_{0}$, $\sigma_{D}=0.01l_{0}$, and $v_{0}=50l_{0}\omega$.

It is also of interest to study the momentum distribution ρ(k,t). Momentum distribution is given by

(9) $$\rho \left(k,t\right)=|\tilde{\psi }{\left(k,t\right)|}^{2},$$

where $\tilde{\psi }\left(k,t\right)$ is the Fourier transform of ψ(x,t). As at t=0 before release, the real-space wavefunction is $\psi \left(x,0\right)=\psi_{g}\left(x\right)\exp\left(imv_{0}x/\hbar \right)$ (see Equation (3)), and the corresponding initial momentum distribution is then $\rho \left(k,0\right)=|{\tilde{\psi }}_{g}\left(k-mv_{0}/\hbar \right){|}^{2}$, where ${\tilde{\psi }}_{g}\left(k-mv_{0}/\hbar \right)$ is just a *k*-shift from ${\tilde{\psi }}_{g}\left(k\right)$, the Fourier transformation of $\psi_{g}\left(x\right)$. In fact, ${\tilde{\psi }}_{g}\left(k\right)$ is also a Gaussian function. The initial momentum distribution is shown by the red curve in Figuer Figure 3. Figure 3 shows only the case with $V_{D}=50\varepsilon_{0}$. When the system reaches equilibrium ($t\gg t_{\mathrm{th}}$), the numerically solved momentum distribution is shown by the blue curve in Figure 3, featuring two peaks at the high-*k* turning points. When comparing the t=0 and $t\gg t_{\mathrm{th}}$ momentum distributions in Figure 3, it is important to note the following. (i) The case of t=0 is asymmetric which implies a nonequilibrium state; the case of $t\gg t_{\mathrm{th}}$ is symmetric which implies an equilibrium state. (ii) Through the effect of random disorder, initial mechanical energy $E_{0}=mv_{0}^{2}/2$ has been transformed to thermodynamic energy at equilibrium, which is distributed among different *k* states in a wide range up to the cut-off turning points.

## 4. Energy Distribution

**Figure 4:**
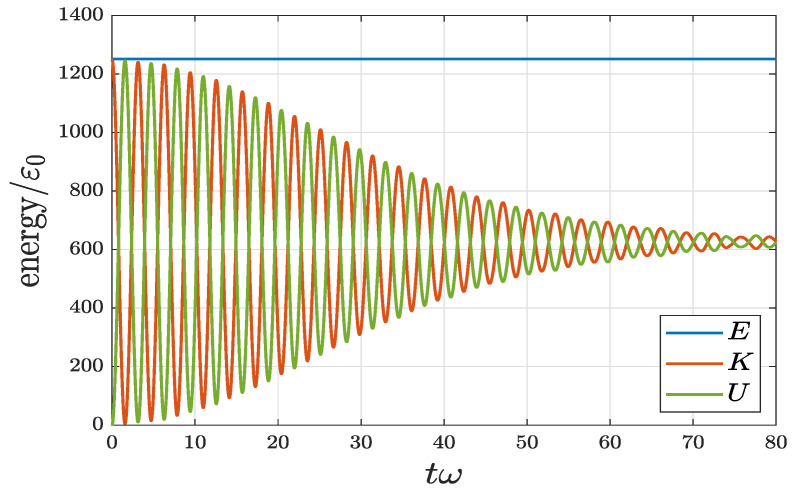
Energy evolution of a quantum harmonic oscillator with random disorder. Total energy is conserved in the whole process and kinetic energy and potential energy associated with harmonic trapping are evenly distributed at equilibrium. The parameters used are $V_{D}=50\varepsilon_{0}$, $\sigma_{D}=0.01l_{0}$, and $v_{0}=50l_{0}\omega$.

In the present system, total energy is the combination of three parts: $E\left(t\right)=K\left(t\right)+U\left(t\right)+V_{\mathrm{dis}}\left(t\right)$, where $K\left(t\right)=\int |\hbar \partial_{x}{\psi |}^{2}dx/\left(2m\right)$ is the kinetic energy, $U=\int \left(m\omega^{2}x^{2}/2\right){\left|\psi \right|}^{2}dx$ is the potential energy associated with harmonic trapping, and $V_{\mathrm{dis}}=\int V_{\mathrm{dis}}\left(x\right){\left|\psi \right|}^{2}dx$ is the potential energy associated with random potential. For random potential, the associated $V_{\mathrm{dis}}$ is typically small compared to *K* and *U*. Figure 4 shows the evolutions of *K* and *U*, as well as the total energy *E* for the case of $V_{D}=50\varepsilon_{0}$, $\sigma_{D}=0.01l_{0}$, and $v_{0}=50l_{0}\omega$.

Unlike the classical damped harmonic oscillator where total energy is dissipated, the total energy *E* of the current disordered quantum harmonic oscillator is conserved. The disorder does not store energy during the thermalization process. It plays the role only to redistribute the energy and result in the final energy distribution. Both *K* and *U* are oscillating at the beginning. When *t* just passes $t_{\mathrm{th}}$, the oscillation becomes relatively smaller, and when $t\gg t_{\mathrm{th}}$, they approach the static limit. As a matter of fact, energy distribution is another indication for the system going from a nonequilibrium to an equilibrium state.

One important feature shown in Figure 4 is that when the system reaches equilibrium, thermodynamic energy *E* is evenly distributed in *K* and *U*. Thus, at equilibrium the virial theorem is satisfied: K=U=E/2. This is analogous to the case for a classical harmonic oscillator, the time-averaged 〈K〉=〈U〉=E/2. Moreover, for the present system, the equilibrium temperature $T_{\mathrm{eq}}$ can be obtained as $k_{B}T_{\mathrm{eq}}/2=K\left(t\gg t_{\mathrm{th}}\right)=U\left(t\gg t_{\mathrm{th}}\right)$. In view of the static value shown in Figure 4, $K\left(t\gg t_{\mathrm{th}}\right)≃U\left(t\gg t_{\mathrm{th}}\right)≃625\varepsilon_{0}$, it is identified that $T_{\mathrm{eq}}≃1250\varepsilon_{0}/k_{B}$.

## 5. Entropy Evolution

Studies of thermalization often follow the von Neumann entropy

(10) $$S_{v}\left(t\right)=-k_{B}\mathrm{Tr}\left[\hat{\rho }\left(t\right)\ln\hat{\rho }\left(t\right)\right],$$

where $\hat{\rho }=\left|\psi \left(t\right)\rangle \langle \psi \left(t\right)\right|$ is the density operator. Thus, the production rate of $S_{v}$ is

(11) $$\frac{dS_{v}\left(t\right)}{dt}=-k_{B}\mathrm{Tr}\left[\dot{\hat{\rho }}\left(t\right)\ln\hat{\rho }\left(t\right)+\dot{\hat{\rho }}\left(t\right)\right],$$

where $\dot{\hat{\rho }}\equiv \partial \hat{\rho }/\partial t$ and the second term in (11) vanishes due to the conservation of total states. It is well known that $S_{v}\left(t\right)$ as well as $dS_{v}\left(t\right)/dt$ are basis-independent, i.e., invariant under unitary transformations. In terms of arbitrary bases,

(12) $$\begin{array}{ccc} S_{v}\left(t\right) & = & -k_{B}\sum_{i,j}\langle i|\hat{\rho }|j\rangle \langle j|\ln\hat{\rho }|i\rangle \\ \frac{dS_{v}\left(t\right)}{dt} & = & -k_{B}\sum_{i,j}\langle i|\dot{\hat{\rho }}|j\rangle \langle j|\ln\hat{\rho }|i\rangle . \end{array}$$

However, if both basis |i〉 and |j〉 are chosen to be the eigenstates of $\hat{\rho }$, then

(13) $$\begin{array}{ccc} \frac{dS_{v}\left(t\right)}{dt} & = & -k_{B}\sum_{i}\langle i|\dot{\hat{\rho }}|i\rangle \langle i|\ln\hat{\rho }|i\rangle \\ & = & \frac{ik_{B}}{\hbar }\sum_{i}\langle i|\left[\hat{H},\hat{\rho }\right]|i\rangle \langle i|\ln\hat{\rho }|i\rangle =0, \end{array}$$

or $S_{v}\left(t\right)$ is constant, which implies von Neumann entropy is not eligible to describe the time direction of an isolated quantum system [41,42].

Alternatively we consider the Shannon entropy [43,44,45]

(14) $$\begin{array}{c} S\left(t\right)=-k_{B}\sum_{i}\langle i|\hat{\rho }\left(t\right)|i\rangle \ln\langle i|\hat{\rho }\left(t\right)|i\rangle , \end{array}$$

where the basis |i〉 is arbitrary but should rule out the eigenstates of either $\hat{\rho }$ or $\hat{H}$. Unlike the basis-independent von Neumann entropy, Shannon entropy is basis-dependent. To acknowledge that the choice is arbitrary, we consider three choices. When the basis |i〉 is chosen to be the position eigenstates, the corresponding Shannon entropy

(15) $$S_{x}\left(t\right)=-k_{B}\int \rho \left(x,t\right)\ln\left[l_{0}\rho \left(x,t\right)\right]dx,$$

where $\rho \left(x,t\right)={\left|\psi \left(x,t\right)\right|}^{2}$ is the real-space density distribution (see also (5)). Alternatively, if the basis |i〉 is chosen to be the momentum eigenstates, the corresponding Shannon entropy is

(16) $$S_{p}\left(t\right)=-k_{B}\int \rho \left(k,t\right)\ln\left[\frac{\rho (k,t)}{l_{0}}\right]dk,$$

where $\rho \left(k,t\right)=|\tilde{\psi }{\left(k,t\right)|}^{2}$ is the momentum distribution (see also (9)).

**Figure 5:**
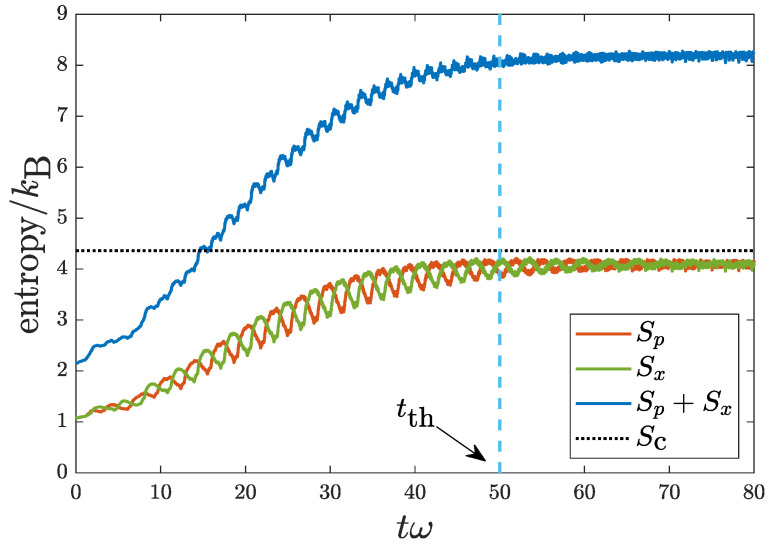
Evolutions of Shannon entropy $S_{x}\left(t\right)$, $S_{p}\left(t\right)$, and the sum $S_{p}\left(t\right)+S_{x}\left(t\right)$ for a quantum harmonic oscillator with random disorder. Black dotted line corresponds to the Shannon entropy for a classical harmonic oscillator in the microcanonical ensemble. Parameters used are $V_{D}=50\varepsilon_{0}$, $\sigma_{D}=0.01l_{0}$, and $v_{0}=50l_{0}\omega$. The thermalization time is identified to be $t_{\mathrm{th}}=50\left(1/\omega \right)$ (see the text).

Figure 5 shows the evolutions of both $S_{x}\left(t\right)$ and $S_{p}\left(t\right)$. Taking into account all possible phase spaces, the result of sum of the two, $S_{x}\left(t\right)+S_{p}\left(t\right)$, is also shown. While the parameters used, $V_{D}=50\varepsilon_{0}$ and $v_{0}=50l_{0}\omega$, are just a numerical trial, they are close to those of a real experimental set-up with $V_{D}=50.9\varepsilon_{0}$ and $v_{0}=37.5l_{0}\omega$ [10]. In addition, we consider a shorter disorder correlation length $\sigma_{D}=0.01l_{0}$. All three cases consistently show the increase of entropy for the thermalization process, and they maximize (saturate) when the system approaches equilibrium. Therefore, the use of Shannon entropy is suitable to describe the thermalization process in the current isolated quantum system. In Figure 5, the black dotted line corresponds to the Shannon entropy for a classical harmonic oscillator in a microcanonical ensemble. It is obtained by substituting the real-space density distribution $\rho_{c}\left(x\right)$ in Equation (8) into $S_{x}$ in (15). In view of Figure 5, the thermalization time in this case after which the system reaches equilibrium can be unambiguously identified to be $t_{\mathrm{th}}=50\left(1/\omega \right)$.

**Figure 6:**
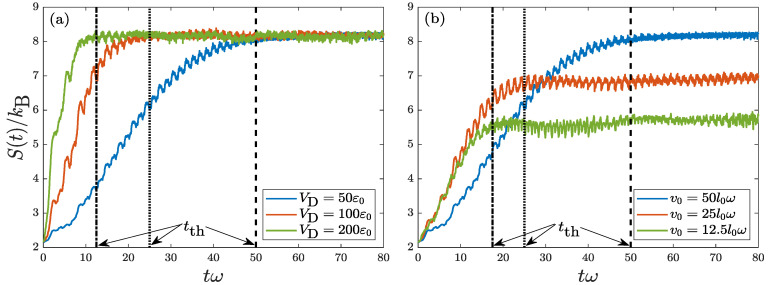
Evolutions of Shannon entropy $S\left(t\right)=S_{x}\left(t\right)+S_{p}\left(t\right)$ for a quantum harmonic oscillator with random disorder. Frame (**a**) considers the effect of disorder potential strength $V_{D}$ with fixed $v_{0}=50l_{0}\omega$ and $\sigma_{D}=0.01l_{0}$. Frame (**b**) considers the effect of initial velocity $v_{0}$ with fixed $V_{D}=50\varepsilon_{0}$ and $\sigma_{D}=0.01l_{0}$. The corresponding thermalization times are identified.

To have a better understanding on how the thermalization time depends on the experimental set-up, in Figure 6 we study the effects of $V_{D}$ and $v_{0}$ on $S\left(t\right)=S_{p}\left(t\right)+S_{x}\left(t\right)$. Figure 6a shows the evolution of S(t) for three disorder strengths: $V_{D}=50\varepsilon_{0}$, $100\varepsilon_{0}$, and $200\varepsilon_{0}$ with fixed $v_{0}=50l_{0}\omega$ and $\sigma_{D}=0.01l_{0}$. The thermalization times for the three cases are identified to be $t_{\mathrm{th}}=50\left(1/\omega \right)$, 25(1/ω), and 12.5(1/ω), respectively. Therefore, the larger the $V_{D}$ is, the faster the system thermalizes. More interestingly, the effect of $V_{D}$ on the thermalization rate ($1/t_{\mathrm{th}}$) is linear, $1/t_{\mathrm{th}}\propto V_{D}$. The linear behavior seems to be quite general in all regimes (noninteracting and interacting) in an isolated quantum system.

Figure 6b shows the evolution of S(t) for three initial velocities: $v_{0}=50l_{0}\omega$, $25l_{0}\omega$, and $12.5l_{0}\omega$ with fixed $V_{D}=50\varepsilon_{0}$ and $\sigma_{D}=0.01l_{0}$. The thermalization times for the three cases are found to be $t_{\mathrm{th}}=50\left(1/\omega \right)$, 25(1/ω), and 17(1/ω), respectively. Thus, $t_{\mathrm{th}}$ goes monotonically with $v_{0}$. In an interacting Bose condensate with disorder, it has been found that when $v_{0}>c$ (*c* being the sound velocity), thermalization will develop, while when $v_{0}\leq c$, thermalization is hardly developed or will not develop [46]. It thus suggested in an interacting system that the thermalization is intimately related to the generation of elementary excitations [10]. In the present noninteracting system, in contrast, the situation is very different. There are only free real particles without the quasiparticles. Due to the effect of disorder, the system tends to thermalize faster with a smaller $v_{0}$ as in this case lesser initial mechanical energy needs to be transformed to the thermodynamic one.

To check the applicability of the Shannon entropy, we also consider the basis of the generalized coherent states, |i〉=|α,n〉, where [47]

(17) $$\langle x|\alpha ,n\rangle =\frac{1}{2^{\frac{n}{2}}\sqrt{n!}\pi^{\frac{1}{4}}\sqrt{l_{0}}}\exp\left[-\frac{{\left(x-\bar{x}\right)}^{2}}{2l_{0}^{2}}\right]H_{n}\left(\frac{x-\bar{x}}{l_{0}}\right)\exp\left\{i\left[-\left(n+\frac{1}{2}\right)\omega t+\frac{x\bar{p}}{\hbar }-\frac{\bar{x}\bar{p}}{2\hbar }\right]\right\}.$$

Here, $H_{n}\left(x\right)$ are Hermite polynomials, $\bar{x}=\langle \alpha ,n|\hat{x}|\alpha ,n\rangle =\sqrt{2}l_{0}\left|\alpha \right|\cos\left(\omega t-\theta \right)$, $\bar{p}=\langle \alpha ,n|\hat{p}|\alpha ,n\rangle =-(\sqrt{2}\hbar |\alpha |/l_{0})\sin\left(\omega t-\theta \right)$, and θ is some arbitrary phase. The corresponding Shannon entropy is given by

(18) $$S_{\alpha }\left(t\right)=-k_{B}\sum_{n=0}^{\infty }\langle \psi \left(t\right)|\alpha ,n\rangle \langle \alpha ,n|\psi \left(t\right)\rangle \ln\left[\langle \psi (t)|\alpha ,n\rangle \langle \alpha ,n|\psi (t)\rangle \right].$$

**Figure 7:**
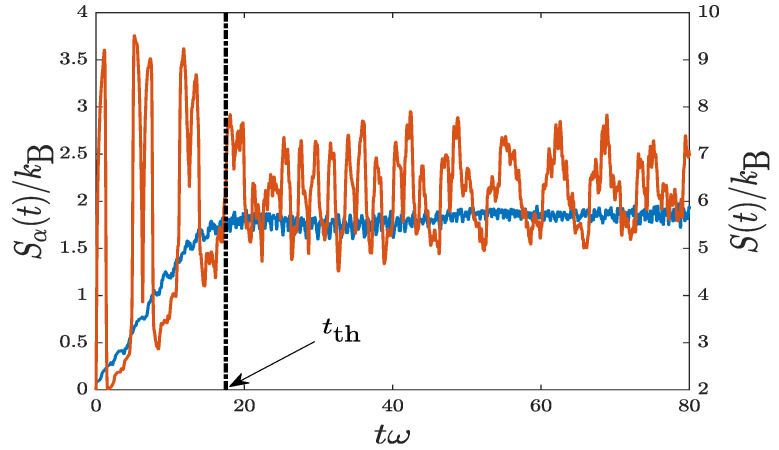
Evolutions of Shannon entropy $S_{\alpha }\left(t\right)$ for a quantum harmonic oscillator with random disorder (red curve). The parameters are $v_{0}=12.5l_{0}\omega$, $V_{D}=50\varepsilon_{0}$, and $\sigma_{D}=0.01l_{0}$. As the FPUT recurrence effect is significant, the key is to follow local minima of $S_{\alpha }\left(t\right)$. For comparison, S(t) (blue curve) is also shown for the same set of parameters (see also in Figure 6b). The thermalization time is identified to be $t_{\mathrm{th}}≃17\left(1/\omega \right)$, consistent in both cases.

For simplicity, for this basis we choose the case with t=0, $\bar{x}=0$, and $\bar{p}=\sqrt{2}\hbar \left|\alpha \right|/l_{0}\equiv mv_{0}$. In this case, Equation (17) results in

(19) $$\langle x|\alpha =mv_{0}l_{0}/\sqrt{2}\hbar ,n=0\rangle \to \frac{1}{\pi^{\frac{1}{4}}\sqrt{l_{0}}}\exp\left(-\frac{x^{2}}{2l_{0}^{2}}\right)\exp\left(i\frac{mv_{0}x}{\hbar }\right)$$

which is identical to our initial wavefunction shown in (3). Thus, the initial entropy is expected to be zero in the case of a single basis function. Figure 7 shows the simulation result of $S_{\alpha }\left(t\right)$ with $v_{0}=12.5l_{0}\omega$. We only show the case of relatively smaller $v_{0}$, as it takes much longer computing time to calculate the cases of higher $v_{0}$. For comparison, we also show S(t), which was already shown in Figure 6b.

While the Fermi–Pasta–Ulam–Tsingou (FPUT) recurrence effect [48,49] for the current oscillating system is significant, the maximization process of $S_{\alpha }\left(t\right)$ is still evident. The key is to follow local minima of $S_{\alpha }\left(t\right)$. Of more importance, the thermalization time is predicted to be $t_{\mathrm{th}}≃17\left(1/\omega \right)$, consistent with that predicted in S(t). This concludes that Shannon entropy is an eligible one to study the thermalization in an isolated quantum system.

## 6. Conclusions

In conclusion, we propose a simple, possible scheme to study the thermodynamics in a quantum harmonic oscillator with random disorder. We have numerically shown that due to the effect of random disorder, the system can undergo from nonequilibrium to equilibrium state, i.e., thermalization. During the thermalization process, total energy of the system is conserved and at equilibrium the kinetic and potential energies are evenly distributed. Shannon entropy in different bases are studied and shown to maximize during the thermalization process.
